# Divergent Isoprenoid Biosynthesis Pathways in *Staphylococcus* Species Constitute a Drug Target for Treating Infections in Companion Animals

**DOI:** 10.1128/mSphere.00258-16

**Published:** 2016-09-28

**Authors:** Ana M. Misic, Christine L. Cain, Daniel O. Morris, Shelley C. Rankin, Daniel P. Beiting

**Affiliations:** a Department of Pathobiology, School of Veterinary Medicine, University of Pennsylvania, Philadelphia, Pennsylvania, USA; b Department of Clinical Studies, School of Veterinary Medicine, University of Pennsylvania, Philadelphia, Pennsylvania, USA; University of Nebraska Medical Center

**Keywords:** *Staphylococcus*, companion animals, comparative genomics, fosmidomycin, isoprenoid biosynthesis, One Health

## Abstract

*Staphylococcus* species are a leading cause of skin and soft tissue infections in humans and animals, and the antibiotics used to treat these infections are often the same. Methicillin- and multidrug-resistant staphylococcal infections are becoming more common in human and veterinary medicine. From a “One Health” perspective, this overlap in antibiotic use and resistance raises concerns over the potential spread of antibiotic resistance genes. Whole-genome sequencing and comparative genomics analysis revealed that *Staphylococcus* species use divergent pathways to synthesize isoprenoids. Species frequently associated with skin and soft tissue infections in companion animals, including *S. schleiferi* and *S. pseudintermedius*, use the nonmevalonate pathway. In contrast, *S. aureus*, *S. epidermidis*, and *S. lugdunensis* use the mevalonate pathway. The antibiotic fosmidomycin, an inhibitor of the nonmevalonate pathway, was effective in killing canine clinical staphylococcal isolates but had no effect on the growth or survival of *S. aureus* and *S. epidermidis*. These data identify an essential metabolic pathway in *Staphylococcus* that differs among members of this genus and suggest that drugs such as fosmidomycin, which targets enzymes in the nonmevalonate pathway, may be an effective treatment for certain staphylococcal infections.

**IMPORTANCE** Drug-resistant *Staphylococcus* species are a major concern in human and veterinary medicine. There is a need for new antibiotics that exhibit a selective effect in treating infections in companion and livestock animals and that would not be used to treat human bacterial infections. We have identified fosmidomycin as an antibiotic that selectively targets certain *Staphylococcus* species that are often encountered in skin infections in cats and dogs. These findings expand our understanding of *Staphylococcus* evolution and may have direct implications for treating staphylococcal infections in veterinary medicine.

## Abstract (precis)

Drug-resistant *Staphylococcus* species are a major concern in human and veterinary medicine. There is a need for new antibiotics that exhibit a selective effect in treating infections in companion and livestock animals and that would not be used to treat human bacterial infections. We have identified fosmidomycin as an antibiotic that selectively targets certain *Staphylococcus* species that are often encountered in skin infections in cats and dogs. These findings expand our understanding of *Staphylococcus* evolution and may have direct implications for treating staphylococcal infections in veterinary medicine.

## INTRODUCTION

*Staphylococcus* infections pose a serious health threat to humans, companion animals, and livestock (1–3). *Staphylococcus aureus* is a Gram-positive bacterium that can asymptomatically colonize human skin and the anterior nares, but it is also responsible for mild to severe skin and soft tissue infections and life-threatening endocarditis, pneumonia, and sepsis. The Centers for Disease Control and Prevention estimated that in 2014 there were 72,000 invasive methicillin-resistant *S. aureus* (MRSA) infections in the United States, which resulted in 9,000 deaths (4). Although the prevalence of MRSA carriage in companion animals is low (approximately 0 to 4%) (5, 6) and infections are rare (7), other *Staphylococcus* species are common commensals and pathogens in veterinary medicine (8). *S. schleiferi* and *S. pseudintermedius* are the leading causes of canine and feline skin and ear infections (9); *S. hyicus* causes high-morbidity skin infections in pigs (10) and osteomyelitis in birds (11), while *S. aureus*, *S. agnetis*, and *S. chromogenes* cause mastitis in cattle and are associated with reduced milk quality (5, 12, 13).

The emergence of drug-resistant *Staphylococcus* is a global problem (14–21). Drugs such as erythromycin and cephalexin are commonly used to treat infections in both humans and animals, leading to concern that as resistance to shared antibiotics becomes more widespread, zoonotic transmission of either drug-resistant *Staphylococcus* or horizontal transfer of resistance genes (22) may render these treatments ineffective for both humans and animals. Despite the major animal health burden posed by a range of *Staphylococcus* species, much of our knowledge of *Staphylococcus* biology stems from studies on only a handful species that are important causes of human disease. To address this knowledge gap, we have used whole-genome sequencing of *S. schleiferi* and comparative genomics to identify novel drug targets to treat staphylococcal infections in companion animals.

## RESULTS

### High-quality genome sequences for *S. schleiferi* obtained via long-read sequencing technology.

We previously reported the use of single-molecule, real-time sequencing to generate complete genome sequences of four canine clinical isolates of *S. schleiferi* (23). These isolates represented a spectrum of antibiotic resistance profiles, from susceptible to all tested antibiotics (isolate 2142-05), intermediate resistance (2317-03 and 5909-02), to multidrug resistant (1360-13) (see Table S1 in the supplemental material). The long reads produced by this approach improved the ability to assemble highly repetitive regions of the *S. schleiferi* genome. For example, rRNA operons represent some of the longest repetitive regions in most bacterial genomes (24), and therefore they pose the biggest challenge for assembly. In addition, clustered, regularly interspaced short palindromic repeat (CRISPR) loci, a type of bacterial adaptive immunity, also pose a challenge to assembly, as they are comprised of a variable number of short, repetitive sequence elements. Our sequencing generated numerous long reads that completely spanned both of these difficult-to-assemble regions of the bacterial genome (Fig. S1a and b). Taken together, these data show that high-quality finished genomes have been generated for this important veterinary pathogen.

10.1128/mSphere.00258-16.1 Figure S1  Long sequence reads assisted in assembling long repetitive regions. (a) Long reads generated by single-molecule real-time sequencing span a 5-kb rRNA operon and nearby coding regions. Red, yellow, and blue bars represent rRNA genes, tRNA genes, and protein-coding sequences, respectively. Black and gray lines indicate sequences that map to strain 5909-02 in the forward and reverse direction, respectively (total coverage in this region is ~90×). (b) Alignment of reads in the clustered regularly interspaced short palindromic repeat (CRISPR) region. Blue bars and pink triangles represent protein-coding *cas* genes and 36-bp CRISPR repeat sequences, respectively. Black and gray lines indicate sequences that map across the entire CRISPR repeat-spacer region in strain 1360-13 in the forward and reverse direction, respectively (total coverage in this region is ~80×). The genomes are ordered from smallest number of repeat sequences (4; strain 2142-05) to largest (36; strain 1360-13). Download Figure S1, PDF file, 0.8 MB. Copyright © 2016 Misic et al. 2016 Misic et al. This content is distributed under the terms of the Creative Commons Attribution 4.0 International license.

10.1128/mSphere.00258-16.2 Table S1  Antimicrobial susceptibility and metadata for *S. schleiferi* strains (the *S. schleiferi* strain metadata are shown, including date of collection, body site, and antibiotic susceptibility profiles; the antibiotic susceptibilities were determined using the Microscan Walkaway 40 PC20 Gram-positive combo-panel [Dade Behring, Sacramento, CA, USA]). Download Table S1, DOCX file, 0.02 MB. Copyright © 2016 Misic et al. 2016 Misic et al. This content is distributed under the terms of the Creative Commons Attribution 4.0 International license.

### Comparative genomic analysis of staphylococcal species.

**FIG 1:**
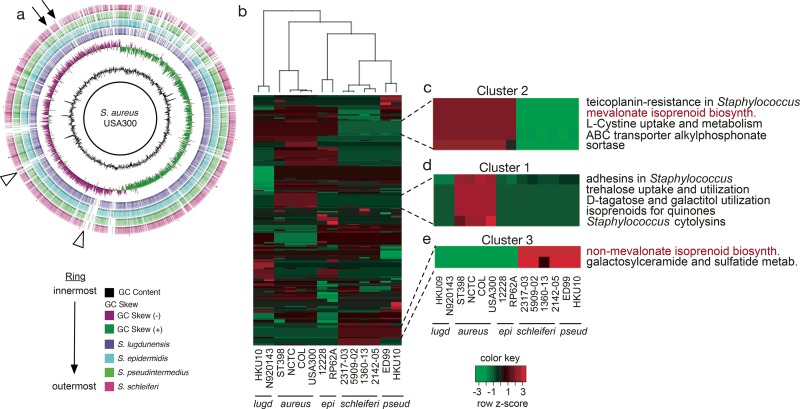
Comparative genomic analysis of *Staphylococcus* genomes. (a) Circular alignment of whole-genome sequences for five *Staphylococcus* species. Working from the inner-most ring out: *S. aureus* subsp. *aureus* USA300 (solid black ring); GC content of *S. aureus* USA300; GC skew of *S. aureus* USA300; *S. lugdunensis* (purple); *S. epidermidis* (blue); *S. pseudintermedius* ED99 (green); *S. schleiferi* strain 1360-13 (red). Arrows indicate genomic regions missing from canine-associated *Staphylococcus*. Triangles point to regions missing from all strains examined but which were present in *S. aureus* USA300. (b) A hierarchical clustering heat map of differentially abundant subsystems among 14 selected *Staphylococcus* genomes from five species. (c) Five selected subsystems found in higher abundance in *S. aureus*, *S. lugdunensis*, and *S. epidermidis* than in *S. pseudintermedius* or *S. schleiferi*. (d) Five selected subsystems found in higher abundance in *S. aureus* than in all the other species examined. (e) Two selected subsystems found in higher abundance in canine-associated *Staphylococcus* species (*S. schleiferi* and *S. pseudintermedius*) than in human-associated species (*S. aureus*, *S. epidermidis*, and *S. lugdunensis*). In panels c, d, and e, only subsystems for which at least two genes were different between the compared groups are shown. The red text in panels c and e highlights isoprenoid biosynthesis pathways.

To better understand the population genetics of *Staphylococcus* and identify putative drug targets, we compared our *S. schleiferi* genomes with publicly available complete genome sequences for four other *Staphylococcus* species. These included *S. pseudintermedius*, a leading cause of companion animal infections, and *S. epidermidis*, *S. lugdunensis*, and *S. aureus* USA300, important causes of community-acquired infections in humans. Genomes were aligned to strain USA300 via BLAST v2.2.22 (25) and visualized with Brig v0.95 (26) (Fig. 1a). A visual inspection of the circular alignments revealed genomic regions that were present in the human-associated *S. aureus* strain USA300 but absent from canine-associated species (Fig. 1a, arrows). Similarly, other regions were absent from all species examined except for *S. aureus* USA300 (Fig. 1a, arrowheads). To more robustly identify genetic pathways differentially abundant among *Staphylococcus* species, we expanded our analysis to include two to four strains for each of the five species shown in Fig. 1a. In total, genomes from 14 strains were annotated by using Rapid Annotation through Subsystems Technology (RAST) (27), allowing gene membership for 382 subsystems to be compared (see Table S2 in the supplemental material). A total of 147 subsystems were conserved, with an equal number of genes present in each subsystem across all 14 strains examined. In contrast, genes in 235 subsystems were differentially abundant among two or more species.

10.1128/mSphere.00258-16.3 Table S2  RAST-annotated subsystems of 14 *Staphylococcus* genomes (from a total of 382 genomes, 147 subsystems were conserved, with an equal number of genes present in each subsystem across all 14 strains examined; genes in 235 subsystems were differentially abundant among two or more species). Download Table S2, XLSX file, 0.03 MB. Copyright © 2016 Misic et al. 2016 Misic et al. This content is distributed under the terms of the Creative Commons Attribution 4.0 International license.

To identify relationships within the subsystems data, hierarchical clustering was performed to group bacterial strains based on similar subsystems profiles (Fig. 1b, top dendrogram), as well as to group subsystems (Fig. 1b, rows) that were enriched in canonically human-associated species (*S. aureus*, *S. epidermidis*, and *S. lugdunensis*) or canine-associated species (*S. schleiferi* and *S. pseudintermedius*). As expected, strains from the same species clustered most closely together (Fig. 1b, top dendrogram). The human- and canine-associated *Staphylococcus* species segregated, indicating that they differ in their functional gene content.

### Differences in virulence factors and metabolic pathways dominate subsystems analysis.

At least three distinct subsystem clusters emerged from our analysis. Cluster 1 consisted of 15 subsystems enriched in all four *S. aureus* strains but absent from all other species. This subsystem cluster included adhesins and pore-forming cytolysins (Fig. 1d). Given the importance of virulence factors in *Staphylococcus* biology, we examined these subsystems in more detail. *S. aureus* isolates contained more than 20 adhesins, while *S. epidermidis* and *S. lugdunensis* isolates had between 6 and 8 adhesins and the *S. schleiferi* and *S. pseudintermedius* isolates contained 9 to 10 adhesins. Table S3 in the supplemental material includes a list of 23 adhesins and their presence or absence among 14 genomes. One adhesin, staphylocoagulase, activates prothrombin to coagulate blood. Although *S. pseudintermedius* and *S. schleiferi* subsp. *coagulans* (*S. schleiferi* strain 1360-13) can coagulate blood, our subsystems analysis showed that there were no staphylocoagulase genes (*coa*) present in the genomes (see Table S3). Consistent with previous studies of *S. aureus*, we found that *S. aureus* contains more virulence factors than other *Staphylococcus* species (28–30). Table S3 shows select adhesins and antimicrobial resistance factors found among the staphylococcal genomes. A surprising finding was that *S. schleiferi* 1360-13 is methicillin resistant (see Table S1 in the supplemental material), yet a *mecA* or *mecC* gene was not found in the genome nor on a plasmid (see Table S3). A recent report documented a case of an *S. schleiferi* human clinical isolate that was resistant to methicillin yet was penicillin binding protein 2 negative (31). The basis of methicillin resistance in these isolates is not yet understood.

10.1128/mSphere.00258-16.4 Table S3  Select adhesin and antimicrobial resistance genes found among 14 *Staphylococcus* genomes (the presence or absence of selected virulence genes in the respective genomes is indicated with a + or − symbol). Download Table S3, XLSX file, 0.02 MB. Copyright © 2016 Misic et al. 2016 Misic et al. This content is distributed under the terms of the Creative Commons Attribution 4.0 International license.

Cluster 2 comprised 15 subsystems that were absent from canine-associated *Staphylococcus* isolates but which were present across all other species examined (Fig. 1c). Among these subsystems was the mevalonate pathway for isoprenoid biosynthesis. This finding was surprising, given that isoprenoids are an essential class of natural products and that staphylococci have been shown to use the mevalonate pathway for isoprenoid biosynthesis (32, 33). Further examination of the canine-associated *Staphylococcus* species (cluster 3, 13 subsystems) showed that these species use the nonmevalonate pathway, an alternative route to isoprenoid biosynthesis (Fig. 1e). Taken together, these data suggest that staphylococcal species use different routes to generate isoprenoids.

### Fosmidomycin selectively kills bacteria associated with veterinary skin and ear infections.

**FIG 2:**
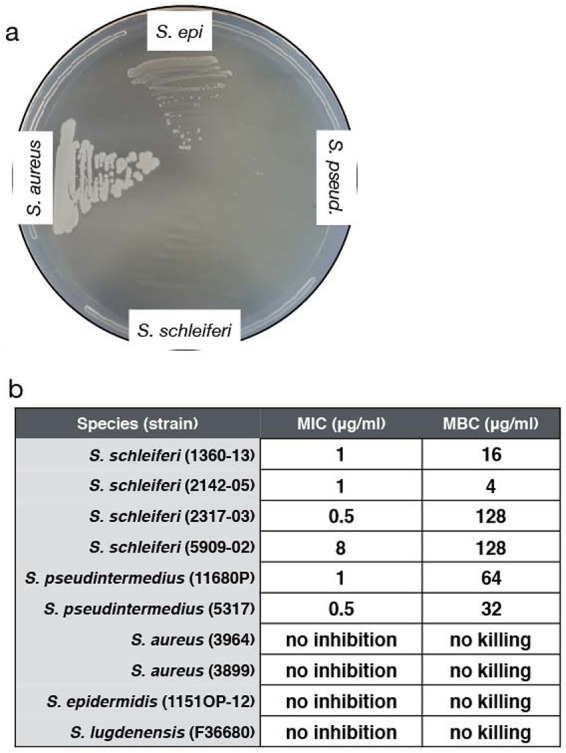
Fosmidomycin selectively kills *Staphylococcus* species associated with companion animal infections. (a) A Mueller-Hinton agar plate containing 50 µg/ml fosmidomycin was streaked with overnight cultures of strains *S. aureus* 3964 (USA100), *S. epidermidis* (1151OP-12), *S. pseudintermedius* (11680P), and *S. schleiferi* (1360-13). (b) MIC and MBC values for 10 *Staphylococcus* strains.

Isoprenoid biosynthesis is a highly conserved and essential process in bacteria, eukaryotes, and plants (33, 34), and inhibitors have been used to target both the mevalonate and the nonmevalonate pathways. We hypothesized that if *S. schleiferi and S. pseudintermedius* used the nonmevalonate pathway to synthesize isoprenoids, they should be sensitive to the drug fosmidomycin, a phosphonic acid derivative that blocks the first committed step of the nonmevalonate pathway via inhibition of 1-deoxy-d-xylulose 5-phosphate reductoisomerase (Dxr) (35). To test this hypothesis, *S. schleiferi* and *S. pseudintermedius* (canine associated) and *S. aureus* and *S. epidermidis* (human associated) were grown on Mueller-Hinton agar plates supplemented with 50 µg/ml fosmidomycin. As expected, *S. aureus* and *S. epidermidis*, both of which are reported to use the mevalonate pathway (33), grew in the presence of fosmidomycin. In contrast, fosmidomycin completely restricted growth of *S. schleiferi* and *S. pseudintermedius* (Fig. 2a)*.* To expand on this analysis, we used MIC and minimum bactericidal concentration (MBC) assays to quantify fosmidomycin activity against a panel of *Staphylococcus* strains from the five species evaluated in Fig. 1. While *S. aureus*, *S. lugdunensis*, and *S. epidermidis* grew normally even in the presence of 256 µg/ml of the drug, *S. schleiferi* and *S. pseudintermedius* were inhibited by fosmidomycin concentrations as low as 0.5 µg/ml and were killed by concentrations of 4 to 16 µg/ml (Fig. 2b). These results provide a biochemical validation of our comparative genomics data (Fig. 1) and suggest that this antibiotic is active against specific *Staphylococcus* species.

### Divergent isoprenoid biosynthesis in the genus *Staphylococcus* is associated with host species.

**FIG 3:**
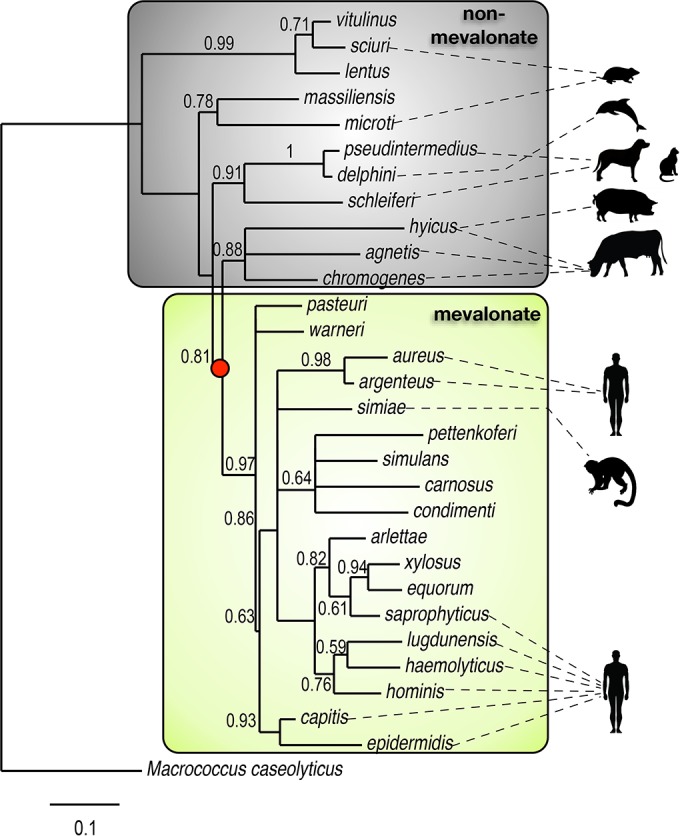
Evolution of isoprenoid biosynthesis in *Staphylococcus*. A maximum-likelihood phylogenetic tree constructed from the *Staphylococcus* superoxide dismutase gene (*sodA*) is shown. Gray and yellow boxes indicate *Staphylococcus* species that utilize the nonmevalonate or mevalonate pathway, respectively. Silhouettes show nonexclusive host associations. The red dot indicates the branch point in the tree where the mevalonate pathway emerged. The tree was rooted with *Macrococcus caseolyticus* as the outgroup.

Although many Gram-negative bacteria have been described to use the nonmevalonate pathway (32, 36, 37), *S. aureus* and other *Staphylococcus* species have long been cited as examples of bacteria that use the mevalonate pathway (32, 33). This prompted us to expand our bioinformatics analysis beyond the five *Staphylococcus* species examined thus far to explore isoprenoid biosynthesis more broadly across the genus. The superoxide dismutase (*sodA*) gene was used to construct a maximum-likelihood phylogenetic tree, and manual curation with KEGG was used to determine which isoprenoid biosynthesis pathway was present in 29 staphylococcal species (Fig. 3). The *sodA* gene has previously been shown to be a good representative of *Staphylococcus* phylogeny (38). This analysis showed that the nonmevalonate pathway was frequently used by *Staphylococcus* species known to cause disease in companion animals and wildlife (Fig. 3, gray box). Use of the nonmevalonate pathway appears to be an ancestral trait, with use of the mevalonate pathway emerging later (Fig. 3, red dot). *Staphylococcus* species that possess the mevalonate pathway formed a monophyletic group that included many notable human- and primate-associated species, such as *S. hominis*, *S. haemolyticus*, *S. simiae*, and *S. aureus* (Fig. 3, yellow box)*.* Also included in this clade were animal-associated species, including *S. equorum*, *S. gallinarum*, and *S. xylosus*, which are often associated with horses, chickens, and mice, respectively, as well as *S. aureus* strain ST398, which is an important cause of disease in livestock.

**FIG 4:**
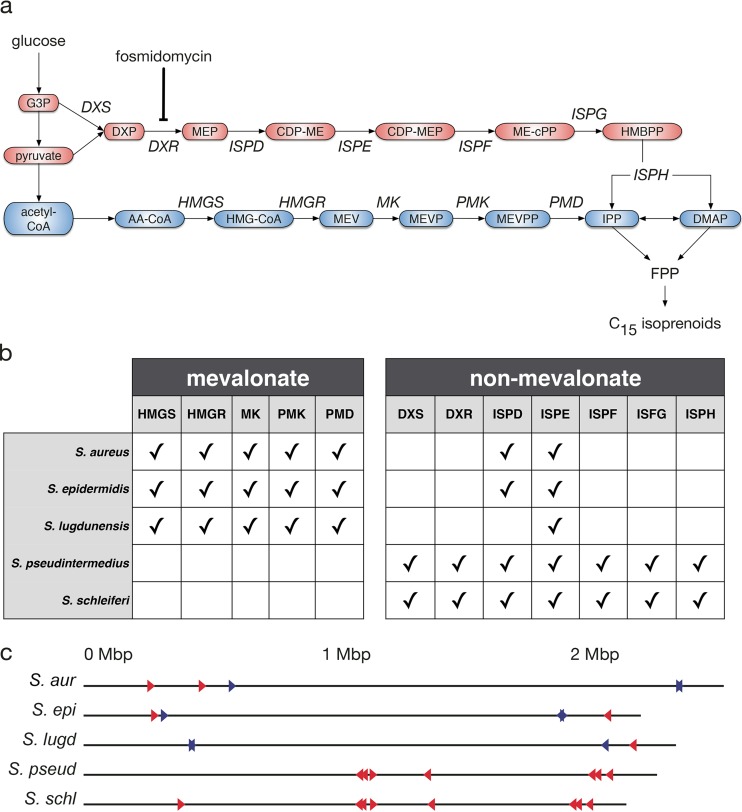
Isoprenoid pathway organization in *Staphylococcus*. (a) Schematic showing enzymes (italics) and intermediates (colored ovals) in the mevalonate (blue) and nonmevalonate (red) pathways. (b) A summary of pathway genes present (indicated by check marks) in *Staphylococcus* species, determined using the KEGG database. (c) Chromosomal location of the mevalonate (blue triangles) and nonmevalonate (red triangles) genes in staphylococcal genomes are shown. The genomes are presented as linear chromosomes oriented such that 0 Mbp is the origin of replication. *S. aur*, *S. aureus*; *S. epi*, *S. epidermidis*; *S. lugd*, *S. lugdunensis*; *S. pseud*, *S. pseudintermedius*; *S. schl*, *S. schleiferi*.

Lateral gene transfer has been suggested to be common in the evolution of isoprenoid biosynthesis in bacteria (37), and this prompted us to examine the gene membership of both pathways for a range of *Staphylococcus* species. The mevalonate pathway begins with acetyl coenzyme A (CoA), which undergoes five sequential enzymatic reactions to generate isopentenyl pyrophosphate (IPP) and dimethylallyl pyrophosphate (DMAPP), which are then converted to isoprenoids (Fig. 4a, blue). In contrast, the nonmevalonate pathway involves a completely distinct set of seven sequential enzymatic steps to convert pyruvate or glucose-3-phosphate to IPP and DMAPP (Fig. 4a, red).

While *S. aureus*, *S. epidermidis*, and *S. lugdunensis* each possess every gene in the mevalonate pathway, they have also retained some nonmevalonate pathway enzymes, including 2-C-methyl-d-erythritol 4-phosphate cytidylyltransferase (IspD) and 4-diphosphocytidyl-2C-methyl-d-erythritol kinase (IspE) (Fig. 4b; see also Table S4 in the supplemental material). In contrast, canine-associated *S. pseudintermedius and S. schleiferi* possess all members of the nonmevalonate pathway and completely lack genes of the mevalonate pathway (Fig. 4b; see Table S4). The genomic location of functionally related genes can reflect their evolutionary history. Similar to the *Staphylococcus* mevalonate pathway (Fig. 4c, red triangles) (33), we found that the nonmevalonate pathway genes were dispersed across the chromosome (Fig. 4c, blue triangles). Taken together, these data argue against a recent lateral transfer of either pathway.

10.1128/mSphere.00258-16.5 Table S4  Isoprenoid biosynthesis genes in 14 *Staphylococcus* genomes (the mevalonate and nonmevalonate pathway gene names, abbreviations, enzyme catalog numbers, and locus identifiers of select *Staphylococcus* species are shown). Download Table S4, XLSX file, 0.05 MB. Copyright © 2016 Misic et al. 2016 Misic et al. This content is distributed under the terms of the Creative Commons Attribution 4.0 International license.

## DISCUSSION

The majority of studies examining the metabolism and biology of *Staphylococcus* have focused on *S. aureus*. While *S. aureus* is an important pathogen in humans and animals, there are many other *Staphylococcus* species of great importance to veterinary medicine. Early biochemical experiments conducted primarily with *S. aureus* helped to establish a dogma that *Staphylococcus* uses the mevalonate pathway to synthesize essential isoprenoids. Our data challenge this dogma and show that isoprenoid biosynthesis differs between *Staphylococcus* species. Given that mammals use the same pathway, it has been assumed that targeting isoprenoid biosynthesis is not a viable strategy to treat staphylococcal bacterial infections in humans or other animals. These data point to inhibitors of the nonmevalonate pathway, such as fosmidomycin, as potential antimicrobials to treat certain *Staphylococcus* infections in animals, in particular in companion animals where species utilizing the nonmevalonate pathway are a major cause of skin and ear infections.

The main target of fosmidomycin is the Dxr protein, but there is evidence that it can also inhibit a downstream enzyme, IspD, *in vitro* and *in vivo* (39). As is the case with any antibiotic, resistance to fosmidomycin could develop in *Staphylococcus*, either by blocking entry or accumulation of the drug in the bacterium or via mutations in the Dxr binding site, both of which have been reported in other pathogens (40–42). Despite these potential problems, the current clinical literature suggests that fosmidomycin could be a promising drug to treat infections caused by *Staphylococcus* species that use the nonmevalonate pathway. Fosmidomycin is extremely well-tolerated and exhibits low toxicity in mammals (32). *Plasmodium falciparum*, the cause of malaria, also synthesizes isoprenoids via the nonmevalonate pathway, and fosmidomycin was shown to be effective in killing the parasite in culture and achieved cure rates of 85 to 100% in clinical trials when administered alone or in combination with clindamycin (43, 44).

Most Gram-negative bacteria synthesize isoprenoids via the nonmevalonate pathway. However, across different bacterial phyla, there are examples of species that use the mevalonate pathway, including *Streptococcus*, *Lactobacillus*, *Myxococcus*, and *Borrelia*. In addition, some *Pseudomonas* species, including *Pseudomonas mevalonii*, are known to use hydroxymethylglutaryl-CoA reductase (the third enzyme of the mevalonate pathway) for degradative functions (45, 46). Only *Listeria monocytogenes* and a few species of *Streptomyces* are known to possess both pathways, but in both organisms the nonmevalonate pathway plays the essential role in primary metabolism (37), while the mevalonate pathway is dispensable (47). Based on our data, *Staphylococcus* constitutes a unique example of a bacterial genus whose species utilize different isoprenoid biosynthesis pathways. Our phylogenetic analysis results (Fig. 3) are consistent with previous evolutionary studies that have suggested that the nonmevalonate pathway is the ancestral pathway in bacteria and the mevalonate pathway was acquired later through lateral gene transfer (34); this is further supported by our finding that deep-branching taxa (such as the *S*. *sciuri* and *S. intermedius* groups) use the nonmevalonate pathway, while the more recently branched taxa (including the *S. aureus* and *S. epidermidis* groups) use the mevalonate pathway.

An outstanding question is why *Staphylococcus* species evolved to use different pathways for isoprenoid synthesis and whether pathway usage influences host range selection. There is abundant literature on virulence factors influencing host range (48), but less is known about the role of bacterial metabolism. Interestingly, among the *Staphylococcus* species we analyzed, mevalonate pathway usage was associated with species that are found across human, nonhuman primate, and animal hosts. In contrast, nonmevalonate pathway usage was only associated with an animal host range. One possible explanation for this observation may lie in the secondary metabolites produced by these pathways and their interaction with the host immune system: 1-hydroxy-2-methyl-2-(E)-butenyl 4-diphosphate (HMB-PP) is an intermediate of the nonmevalonate pathway and a potent activator of Vγ2/Vδ2 (also called Vγ9/Vδ2) T cells (49). HMB-PP has 1,000 times greater immune stimulatory activity than IPP, the analogous intermediate produced by the mevalonate pathway (49, 50). Vγ2Vδ2 cells make up 1 to 5% of peripheral T cells but expand to >50% and rapidly traffic to barrier surfaces in response to pathogens that produce HMB-PP (51, 52), yet these cells are only found in humans and nonhuman primates. This could lead to a scenario in which mevalonate usage (i.e., by *S. aureus*) results in a relatively weak Vγ2Vδ2 signal, thereby allowing colonization of human and nonhuman primate skin. Moreover, spread to animals would not be impeded, because these hosts completely lack the Vγ2Vδ2-bearing cells. In contrast, nonmevalonate pathway usage (i.e., by *S. schleiferi* and *S. pseudintermedius*) would result in production of the potent Vγ2Vδ2 ligand HMB-PP, but this would only be of consequence if the bacteria were on human or nonhuman primate hosts. Thus, one plausible hypothesis is that nonmevalonate pathway usage by *Staphylococcus* may contribute to an animal host range restriction, but this remains to be tested. Interestingly, *S. schleiferi* and *S. pseudintermedius* were first identified in human infections and are occasionally reported to cause serious human disease, but such cases are primarily observed in infants, the elderly, immunocompromised patients, or as a consequence of medical complications (53–57), perhaps lowering the immunological barrier to transmission.

## MATERIALS AND METHODS

### Bacterial strains, media, and growth conditions.

*Staphylococcus* isolates used for biochemical analysis were collected at the Matthew J. Ryan Veterinary Hospital of the University of Pennsylvania. *S. schleiferi* biochemical identification was carried out on a Microscan Walkaway 40 PC20 Gram-positive combo-panel (Dade Behring, Sacramento, CA). Four banked *S. schleiferi* strains were selected for whole-genome sequencing: 1360-13, 2142-05, 2317-03, and 5909-02. Table S1 in the supplemental material contains a full list of *S. schleiferi* strains used in this study and their associated metadata. For biochemical assays, the following clinical isolates were used: *S. aureus* 3964, *S. aureus* 3899, *S. pseudintermedius* 5317, *S. pseudintermedius* 1168OP, *S. epidermidis* 1151OP-12, and *S. lugdunensis* F36680.

### DNA purification and sequencing.

*S. schleiferi* genomic DNA was purified, sequenced, and assembled into complete genomes as previously described (23). Briefly, DNA was extracted from overnight cultures of *S. schleiferi* isolates by using the Qiagen Genomic Tips kit (Valencia, CA, USA). DNA quantity and quality were assessed using a NanoDrop 1000 spectrophotometer (Thermo Scientific, Pittsburgh, PA) and a Qubit fluorometer (Life Technologies, Grand Island, NY, USA). Agarose gel electrophoresis was used to confirm high-molecular-weight DNA (>50 kb) for single-molecule real-time sequencing on a Pacific Biosciences RSII platform. SMRTbell adapters were ligated, and each strain of *S. schleiferi* was sequenced on 1 cell with one 120-min movie. A hierarchical genome assembly process (HGAP) was performed for each strain using the HGAP.3 module (58). The genome was closed using manual refinement.

### Comparative genomics.

For comparative genome analyses, 14 sequences from GenBank were retrieved for the following organisms: *S. schleiferi* 1360-13 (accession number CP009470), *S. schleiferi* 2142-05 (CP009762), *S. schleiferi* 5909-02 (CP009676), *S. schleiferi* 2317-03 (GenBank: CP010309), *S. pseudintermedius* HKU10-03 (NC_014925.1), *S. pseudintermedius* ED99 (NC_017568), *S. lugdunensis* HKU09-01 (CP001837), *S. lugdunensis* N920143 (FR870271.1), *S. epidermidis* ATCC 12228 (NC_004461), *S. epidermidis* RP62A (NC_002976.3), *S. aureus* subspecies *aureus* ST398 (NC_017333), *S. aureus* subspecies *aureus* USA300_FPR3757 (NC_007793), *S. aureus* subspecies *aureus* COL (NC_002951), and *S. aureus* subspecies *aureus* NCTC 8325 (NC_007795) were downloaded from NCBI (ftp://ftp.ncbi.nlm.nih.gov). Five circular, whole genomes were aligned using the BLAST Ring Image Generator (BRIG) version 0.95 (26) and the Basic Local Alignment Search Tool (BLAST, v. 2/2/22) (25). Functional gene categories were determined with the Rapid Annotation using Subsystem Technology (RAST) v. 2.0 (27) and FigFam v. 70 programs. Subsystems with a standard deviation of zero among species were removed. The remaining differentially abundant subsystems were clustered by a Pearson correlation, and the bacterial species were clustered by a Spearman correlation using the hclust function in R (v. 3.2.0) (59) to reveal species-specific subsystem clusters. The cutree function in R was used to identify groups with similar subsystem abundance profiles.

### Antibiotic susceptibility assays.

The MICs of various antimicrobials (amoxicillin-clavulanic acid, ampicillin, cefazolin, chloramphenicol, ciprofloxacin, clindamycin, erythromycin, gentamicin, imipenem, oxacillin, penicillin, rifampin, tetracycline, trimethoprim-sulfamethoxazole, and vancomycin) were tested via broth microdilution on a Microscan Walkaway 40 PC20 Gram-positive combo-panel (Dade Behring, Sacramento, CA). Fosmidomycin MIC assays were performed following clinical laboratory standards (60). Fosmidomycin (Sigma, St. Louis, MO, USA) was filter sterilized with a 0.2-mm filter (Pall Corporation, Ann Arbor, MI, USA) and was serially 2-fold diluted for a concentration range of 40.96 to 0.25 µg/ml. Tubes containing 1 ml Mueller-Hinton broth (Sigma) were inoculated with 5 × 10^5^ CFU/ml. A 50-μl aliquot of the appropriate fosmidomycin concentration was added to each tube containing the starter culture and was incubated at 37°C overnight with shaking at 250 rpm for 18 to 20 h. The MICs were determined by visual inspection. The MBC was determined by plating 200 µl of the drug-treated cultures onto Mueller-Hinton agar plates (Remel, Lenexa, MA, USA). The lowest concentration at which there was no growth after a 24-h incubation at 37°C was determined to be the MBC. For the plate-based fosmidomycin growth assay, overnight cultures of *Staphylococcus* species grown in Mueller-Hinton broth were subcultured to Mueller-Hinton agar plates containing 50 µg/ml fosmidomycin and were incubated for 24 h at 37°C. All assays were performed and the results were interpreted using Clinical and Laboratory Standards Institute guidelines (60).

### Phylogenetic tree construction and metabolic pathway comparisons.

Gene sequences of *sodA* from 29 staphylococcal species were downloaded from NCBI. The Web-based tool Phylogeny.fr (61) was used to construct a phylogenetic tree from a *sodA* gene multiple-sequence alignment, and *Macrococcus caseolyticus* was set as the outgroup. Sequences were aligned with Muscle v3.7 (62), poorly aligned regions were removed using Gblocks v0.91b (63), and the phylogenetic tree was reconstructed using the maximum likelihood method implemented in PhyML v3.0 (64). Tree rendering was performed using TreeDyn v198.3 (65), and bootstrap values are indicated on the branches. Metabolic pathway reconstructions of each strain were compared *in silico* using the terpenoid backbone biosynthesis pathway from KEGG (66, 67).
