# Supplementary material for: Divergent Isoprenoid Biosynthesis Pathways in Staphylococcus Species Constitute a Drug Target for Treating Infections in Companion Animals
Source: mSphere. 2016 Sep 28;1(5):e00258-16. doi: 10.1128/mSphere.00258-16 (PMC5040788; doi:10.1128/mSphere.00258-16)
Supplement: Figure S1 [file sph005162151sf1.pdf]

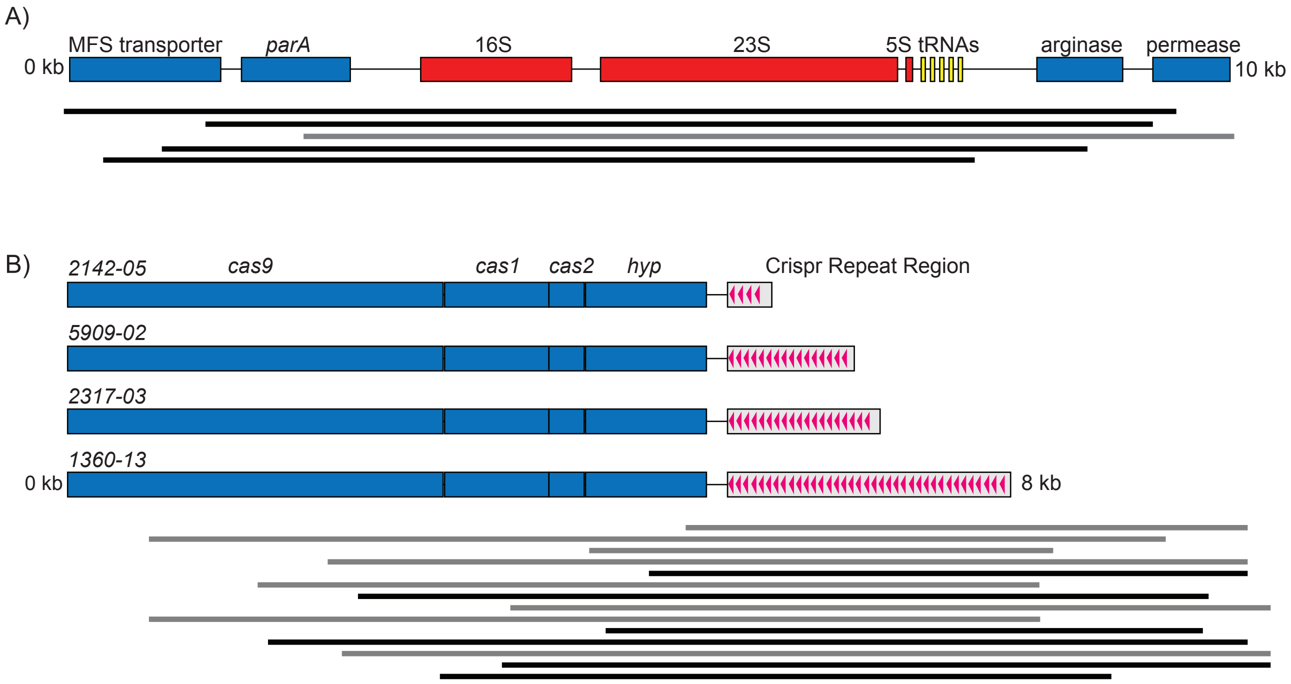

Figure 1: Long sequence reads assisted in assembling long repetitive regions. A) PacBio long reads span a 5 kb rRNA operon and nearby coding regions. Red, yellow and blue rectangles represent rRNA genes, tRNA genes, and protein-coding sequences, respectively. Black lines indicate sequences that map to the region in the forward direction; grey lines indicate sequences that map to the strain 5909-02 region in the reverse direction (total coverage in this region is ~90X). B) Alignment of the CRISPR-cas loci. Blue, grey, and pink rectangles represent protein coding cas genes, CRISPR repeat region, and the 36-bp CRISPR repeat sequences, respectively. Black lines indicate sequences that map across the entire CRISPR repeat-spacer region in strain 1360-13 in the forward direction; grey lines indicate sequences that map in the reverse direction (total coverage in this region is ~80X). The genomes are ordered from smallest (4; strain 2142-05) to largest (36; strain 1360-13) number of repeat sequences.
