# Supplementary material for: Divergent Isoprenoid Biosynthesis Pathways in Staphylococcus Species Constitute a Drug Target for Treating Infections in Companion Animals
Source: mSphere. 2016 Sep 28;1(5):e00258-16. doi: 10.1128/mSphere.00258-16 (PMC5040788; doi:10.1128/mSphere.00258-16)
Supplement: Table S1 [file sph005162151st2.docx]

**Supplemental Table S1**

| ***Organism*** | **1360-13** | **2142-05** | **5909-02** | **2317-03** |
| --- | --- | --- | --- | --- |
| Source | Skin Pyoderma | Ear (otitis) | Skin Pyoderma | Skin Pyoderma |
| Date obtained | 5/14/13 | 5/3/05 | 12/16/02 | 5/21/03 |
| ***Biochemical Test Results:*** |  |  |  |  |
| Coagulase | + | - | - | - |
| Urease | + | + | + | + |
| Beta-lactamase | + | - | + | + |
| ***Antimicrobial susceptibility Results:*** |  |  |  |  |
| Ampicillin | R | S | R | R |
| Amoxicillin-clavulanic acid | R | S | R | R |
| Chloramphenicol | I | S | S | S |
| Clindamycin | R | S | R | S |
| Cefazolin | R | S | R | R |
| Ciprofloxacin | R | S | R | R |
| Erythromycin | R | S | R | S |
| Gentamicin | I | S | S | I |
| Imipenem | R | S | R | R |
| Oxacillin | R | S | R | R |
| Penicillin | R | S | R | R |
| Rifampin | S | S | S | S |
| Trimethoprim-sulfamethoxazole | R | S | S | S |
| Tetracycline | R | S | S | S |
| Vancomycin | S | S | S | S |
